# Teplitz Mineral Water

**Published:** 1893

**Authors:** Robert Newman

**Affiliations:** New York City


### TEPLITZ MINERAL WATER.

Because of my advice "drink Teplitz," I frequently am asked, what is Teplitz, where is Teplitz? Now, dear reader, you know all about it; but I must as a faithful reporter describe in regular order its geographical location and other particulars.

Teplitz-Schönau is one of the oldest and best known watering-places in Europe, situated in Bohemia, not far from Carlsbad. It is frequented yearly by over 32,000 visitors, among whom are crowned heads, principally the Austrian Emperor and his court, as well as other reigning sovereigns. The population proper of Teplitz has according to a late census 20,133 inhabitants.

### POSITION, CLIMATE AND HISTORY.

The beautiful watering place of Teplitz-Schönau is situated in the 50° 38′ 16″ northern latitude, and the 31° 29′ 41″ eastern longitude from Ferro, 718 feet above the level of the Adriatic Sea, in the north-west part of Bohemia, in the Biela Valley, which is as celebrated for its luxuriant and abundant vegetation as for its natural beauties of scenery.

The Erz mountains on the north, belonging to the primary formation, consist chiefly of gneiss, porphyr and granite, and form a natural wall against the raw, cold north wind.

To the south and east stretch the long rides and promontories—the characteristic signs of basaltic formation—of the Mittelgebirge, which thus shields the town against the injurious hot winds of the south. The climate is mild and healthy, the average temperature for the year being 50° F.; a sojourn here is therefore strongly to be commended to those who are entering into a state of convalescence after long illnesses. Teplitz is the oldest watering place in Bohemia and was known and used as such by the Romans. Later historical traditions take, however, a more decided form. Wladislaw I., King of Bohemia, founded a monastery here between 1150 and 1170. Both he and Johanna, the consort of King George of Podebrad, patronized the watering place. In the year 1570, the poet Thomas Mitis celebrated in Latin verse the praises of the place. A great service was rendered to this watering place in 1578 by Wolf, and Bernhard von Wresowitz, who fitted up some baths. In the year 1585 there were already 14 baths. The story has been handed down from that century, that even from the earliest times Teplitz was visited by sick persons, who frequently came from a considerable distance.

Thus the fame of this watering place has gone on increasing up to the present time, emperors and kings, statesmen and savants have hurried to its springs, and there found a cure for their sufferings and debility. Not without justice has Teplitz been called the "Warrior's Bath," since thousands of warriors have here found healing for their wounds. Teplitz-Schönau is a post and telegraph station, as well as the station of the Aussig-Teplitz and Dux-Bodenbach railways. The journey may either be made via Berlin and Dresden, via Cologne, Aschaffenburg, Eger, or via Leipzig, Dresden and Bodenbach.

Teptlitz-Schönau has different springs, cold and warm, besides Moore baths which belong to the alkaline-saline class. The best known spring is the Teplitz Stadtquelle, which is the property of the city of Teplitz. This water has been imported to America, can be bought in New York, and is exclusively spoken of in this article.

The chemical analysis of the *Teplitz Water* is as follows: 10,000 grains of this water contain Solids (ingreius).

| | By Sonnenschein. | By Gintl. |
|---|---|---|
| Sulphate of Potassium........... | 0.228 | 0.171 |
| " " Calcium ............ | 0.560 | L.757 |
| Chloride of Sodium.............. | 0.629 | 0.661 |
| Phosphate of Sodium............ | 0.017 | 0.005 |

| | | |
|---|---|---|
| Carbonate of Sodium | 4.143 | 4.628 |
| " " Calcium | 0.691 | 0.016 |
| " " Lithium | 0.005 | 0.001 |
| " " Strontium | L.021 | 0.002 |
| " " Magnesium | 0.114 | 0.133 |
| " " Manganesium | 0.018 | 0.002 |
| " " Oxide of Iron | 0.155 | 0.004 |
| Fluoride of Calcium | 0.017 | ..... |
| Silicic acid | 0.475 | 0.461 |
| Argillaceous earth | | 0.001 |
| Humine substances | | 0.077 |

GASES.

| | | |
|---|---|---|
| Carbonic acid (as bicarbonates) | 1110.477 | 1011.79 |
| do. (free) | 34.120 | 396.04 |
| Oxygenium | 18.360 | 43.03 |
| Nytrogenium | 50.940 | 104.62 |

The many saline, acidulous and gaseous ingredients shown, lead to the conclusion that the water, before reaching the surface, had broken, with great difficulty, through resisting rocks from great depths, yet it is difficult to determine whether its temperature of 115° F. was created by chemical, on its probably long passage, or through volcanic influences ; it appears however that the substantial qualities of the water are very delicate and plentiful, and that in the copiousness of its ingredients, there are found some gaseous and solid materials which, according to physiological experience, are acknowledged as important factors in the act of digestion and subsistence.

It will be perceived from the analysis that the contents of the water are very similar to the saliva and gastric juice of the human body, and the inference is, that the water is very useful in diseases of the throat and stomach.

*The Physiological action* of this water has been particularly well described by Professor H. Gerold, M. D., privy counsellor of the court, from whom the following is quoted :

The food taken into our bodies, is divided (through the normal process of digestion, which is in part mechanical and partly of a chemical nature), in such manner, that the portion which is essential to life and health is retained in the system, and that portion not required for our well-being is removed.

The substances serving for the subsistence of men are derived partly from organic and partly from inorganic nature.

To the former (organic) belong:

(*a*) The glairy matters (albumen, fibrine, caseine), found in the animal and vegetable kingdoms.

(*b*) The fat, especially that from the animal kingdom (as in milk and butter, and that of the sebacic acids formed through the decomposition of the same).

(*c*) The hydrates of carbon, viz: starch, dextrine, glucose; in short, those substances which assist in the process of digestion. To the latter (inorganic) belongs:

Water (which we require in great quantity, not only to quench thirst and to dissolve food, but as a "provision of life"), the value of which is governed by the mineral substances, gases, salts and alkalis it possesses.

In respect to this property or quality: *i. e.*, as an important "provision of life," let us examine the product of the city spring Teplitz, Bohemia, as a water especially adapted for table use.

There are many other similar opinions on record, all proving that Teplitz water is a healthy table water, agreeable as a pleasant drink for the well.

*The Therapeutical action* of Teplitz water has been described by many—and testimonials of reputable well known physicians are on file in abundance, that the water has benefited and cured many different diseases.

The writer does not wish to quote such diseases as cured, as it may mislead the laymen as well as the profession. Each case and patient needs the full consideration, and if the action and quality of the water is understood, an intelligent physician will know when to order the therapeutical measure.

Medical Counsellor, C. F. Kunze, M. D., has given an elaborate report of cases cured by Teplitz under his direction. The histories of those cases are very interesting for the thinking physician, but for the reasons first stated, they are omitted in this article.

There is a marked difference if patients go to a watering place and are treated there, or if they drink the mineral water at home which has been bottled and exported.

In diseases of the *kidneys and bladder* this water is most effective as a therapeutic agent, it stimulates the kidneys to action, and as long as there is any substance left of sound kidney this water will work and prolong life. I know that in hopeless cases of Bright's disease the patient's life was prolonged one year by the moderate drinking of this water. Care must be taken not to use too much, and as a rule it is not advisable to drink more than one bottle per day.

In diseases of the *bladder* I always order Teplitz ; as an alkaline it will counteract over acidity and allay spasm of the bladder and check the excessive secretion of urates.

In catarrh of the bladder it will effect a cure in most cases, removes pain, irritation, clears the urine of mucous, epithelium and pus corpuscles, and removes the frequent desire of micturition. Such cases require, however, the continuous use of the Teplitz water for months—and in no case can do any harm. Catarrh and other diseases of all mucous membranes will be benefited by this water, such as bronchitis and other affections of the air passages, buccal, nasal cavities, bronchi, lungs, catarrh of the stomach, bowels, etc.

In nervous indigestion, dyspepsia and flatulence the Teplitz is also indicated.

Gout and rheumatism, particularly in its chronic state, are such severe diseases, that the simple drinking of any mineral water cannot effect a cure, but Teplitz will be beneficial. I know cases in which the patients were kept from heavy attacks, and on omission of drinking Teplitz suffered painful relapses. I must warn against certain advertisements, that a certain mineral water will cure gout or rheumatism. I know of no mineral water which will do this to a certainty, without other aids and treatment. Teplitz water may be useful in other ailments, but from experience I can only report as above. As a *table water* I know no better. It is peerless in its purity, palatable and refreshing—a glass of this water in the morning will clear the stomach of accumulated mucous. As a diluent with wines or liquors, or combined with fruit syrups it is very pleasant, and such mixture will keep a clear fluid, and not cause sediments by decomposition.

The Teplitz water is recommended to all epicureans, sick or well.

NEW YORK CITY. ROBERT NEWMAN, M. D.

